# The availability of psychological support following road travel injuries in Namibia: A qualitative study

**DOI:** 10.1371/journal.pone.0258197

**Published:** 2021-10-01

**Authors:** Mitchel Chatukuta, Nora Groce, Jenny Mindell, Maria Kett

**Affiliations:** Research Department of Epidemiology and Public Health, University College London (UCL), London, United Kingdom; Al Mansour University College-Baghdad-Iraq, IRAQ

## Abstract

Road traffic injuries (RTIs) are a major problem worldwide with a high burden of mental health problems and the importance of psychological support following road injury is well documented. However, globally there has been very little research on the accessibility of psychological services following road injury. Namibia is one of the countries most affected by RTIs but no previous studies have been done on this. In this qualitative study we investigated the availability of psychological services to RTI injured in Namibia. Our study findings are in line with those of other global studies in showing inadequate access to psychological support for injury survivors and we discuss the reasons. It is hoped these findings will help policymakers develop ways of enhancing access to psychological support for the many people injured in RTIs in Namibia. The models they develop may also be of use to other LMICs countries with high RTI rates.

## 1.0 Introduction

Road traffic injuries (RTIs) have dramatically risen and are now a major contributor to the global burden of deaths and injuries, with over 1.2 million people killed and between 20 and 50 million injured annually [1]. For every person killed as a result of RTI, at least 20 others sustain non-fatal injuries, and many become permanently disabled [2].

RTIs also have short and long-term psychological outcomes for those affected and their families [3]. Variation in prevalence rates could be due to differences in data collection tools, administration methods and timing of data collection [4], however there is a general consensus that post-injury, the prevalence of psychological disorders is high and may be associated with poorer functional and occupational outcomes [5]. A review of psychiatric morbidity following an RTI showed the most commonly reported disorders were depression (21–67% across studies), anxiety (4–87%), and driving phobia (2–47%), while prevalence of Post-Traumatic Stress Disorder (PTSD) ranged from zero to 100% [6]. Higher prevalence of PTSD has also been identified in RTI survivors as compared with the general population or control groups [7] as well as those injured in RTIs compared to falls [4] Work by [8] established that within the first year post-injury, about 20% of injured survivors developed an acute stress reaction and about 25% displayed psychiatric problems. Long-term psychiatric problems included mood disorder (10%), phobic travel anxiety (20%), and PTSD (11%) [8]. Research by [9] also identified an increase in usage of psychoactive drug consumption in 15% of those with mild-to-moderate injuries and 27% of the severely injured.

Although some studies have found that survivors with more severe injuries experience higher levels of PTSD [7,9,10] argued that severity of road-injury is not always directly proportional to the psychological manifestations and even relatively minor injuries can have profound psychological effects. As an example, a recent study by [11] found that individuals with minor injuries presented with long-term poor mental health related quality of life. Giving another perspective, [12] argued that the emotional and psychological impacts are more widespread and varied than are captured by measures of PTSD or psychiatric illness and they argued for the need of these symptoms to be identified and for injured individuals to be referred to support groups and services.

Other studies have reported that psychological problems following injury can have greater impact on quality of life than the physical injury itself [13,14]. Another strong argument for timely access to mental health support needing to be a priority for RTI survivors is that psychological and emotional wellbeing following injury can compromise productivity. Psychological interventions can also facilitate RTI survivors to return to former levels of productively as well as show improved outcomes regarding health, return to work, and quality of life [15].

However, despite the high burden of mental illness due to RTIs, very little research has been done globally in this subject area and very few studies in particular have been done to investigate the accessibility of psychological services following road injury [16]. The few studies done have largely been undertaken in in high income countries (HICs) and have shown that psychological consequences from RTIs are often neglected by clinical staff treating patients despite the considerable emotional and psychological consequences [17]. For example, in France, [9] found that only 11% of survivors had received psychological help following their injury. Similarly, in the UK, [18] conducted research on injury survivors post-hospitalisation and reported that although some of them felt counselling would have been helpful, they were unable to access this.

The lack of accessibility to services following road injury is likely to be worse in lower and middle income countries (LMICs), which have poorer health infrastructure than HICs. A comprehensive literature review was undertaken as a component of this study and the authors were unable to identify any specific studies on psychological support following RTIs from sub-Saharan Africa (SSA). It is striking that there is so little attention to the subject of psychological support for victims of RTIs from SSA as it is the worst affected region globally in terms of traffic-related injuries, deaths and long-term impairments.

We thus chose to focus our research on this topic. We specifically focused on Namibia, an SSA country with one of the highest RTI injury and death rates globally [1]. According to [19], due to a lack of scientific data collection systems, and formal registry on the prevalence and incidence of mental illnesses in the country, Namibia has considerable challenges with epidemiological data on mental illness. We also focused on Namibia because despite its LMIC status, it is one of five countries in SSA (the others being South Africa, Swaziland, Botswana and Lesotho) that have a system whereby a fuel tax levy Fund has been set up which is meant to provide support for road injury survivors. The fund covers funding for all medical costs, aids and appliances required for RTI victims as well as rehabilitation, and in the event of a serious injury, a lump sum payment and a caregiver allowance [20]. S1 and S2 Appendices provide details of the registration process and benefits available under the scheme. In contrast, in most of the other countries in SSA, motor vehicle third-party liability (MTPL) insurance is used to pay for the health care costs of those injured in crashes, otherwise they have to pay for the costs themselves [21]. With MTPL, the injured survivors, including the driver, can claim compensation from the insurance company to settle their treatment and personal expenditures related to the injury. However the MTPL system has been reported to be highly problematic in a number of countries. Many drivers carry no insurance, despite the fact that they are often legally required to do so, and most pedestrians also carry no insurance. Even where a driver has insurance, drivers, passengers and pedestrians are often unable to get insurance companies to cover even basic injury claims; access to adequate medical care and psychological support services following a road injury are often unavailable or non-existent [21].

This study provides a greater understanding of the levels of psychological services to people injured in RTIs in Namibia and for those not able to and the hindrances that limit them from doing so. We also investigate whether the fuel tax levy’s Motor Vehicle Accident Fund (MVAF) has any impact on the accessibility to what services may exist. It is anticipated these results would be helpful to programme and policy makers in Namibia specifically and more generally could be extrapolated to other countries within and beyond SSA.

## 2.0 Methods

### 2.1 Data collection

This qualitative study was part of a PhD study by the lead author (MC) investigating the long-term impact of road injuries in Namibia and began with a review of the literature described elsewhere in detail [22]. The design of this study was based on the principles of grounded theory research and involved conducting two sets of interviews face to face. The first set, with individuals injured in RTIs in Namibia and the second set, with healthcare workers (HCWs) and disability advocates in Namibia. Both interview sets were conducted via semi-structured questionnaires and included closed questions to elicit information such as “Did you receive any post-trauma psychological care/counselling?” with “yes” or “no” responses, and open-ended questions, where more detailed responses were elicited. An example is: “Following road injury, do survivors receive any psychological support/counselling? All the interviewees preferred to be interviewed in Namibia’s official language, English, although arrangements were in place to use translators if interviewees preferred to be interviewed in Afrikaans or Oshiwambo, the most widely spoken native languages. Please refer to S3 and S4 Appendices for the questionnaires.

### 2.2 Study site and setting

The research was conducted over a five week period at the beginning of February 2017 to mid-May 2017 and the recruitment processes are described in section 2.3. Of the 34 interviews completed, 18 of these were conducted in the capital city, Windhoek, with the rest conducted in other parts of the country, including Rehoboth, Swakopmund, and Walvis Bay to ensure a better understanding of the experience of RTIs nationally.

### 2.3 Sampling methods used

The exploratory nature of this study and access to the population under study necessitated the use of purposive sampling. Road injury victims were accessed via establishing contacts with two organisations, the Namibian Association of People with Physical Disabilities (NAPPD) and the National Federation of People with Disabilities in Namibia (NFPDN). Both organisations were responsive to the need for this study and identified four possible participants. The four individuals were approached via phone by MC, and three agreed to participate whilst one declined. Eleven additional participants were recruited through snowballing from this initial core group, with contact made by phone by MC. In total, the sample consisted of 14 individuals with long-term road traffic injury-related disabilities. Of the 11 individuals recruited through snowball sampling, nine were not aligned to the NAPPD nor to the NFPDN.

MC started recruitment of HCWs via an internet search of specific service units which focused on identifying HCWs involved in caring for RTI victims or those who had insight/influence on this process. Eight HCWs were initially approached directly through an introductory e-mail followed by a phone call and invited to participate in the study. Twelve more HCWs suitable for the study were recruited via snowball sampling, with contact made by phone by MC, and the sample had a total of 20 participants.

### 2.4 Ethical considerations

Ethical approval was granted by both the Biomedical Research Ethics Committee and Research Management Committee of the Ministry of Health and Social Services in Namibia (No. 17/3/3) and the UCL Ethics Committee (No: 7417/001). Participants were given information sheets and assurance about the maintenance of confidentiality throughout the study. Following this, they were asked to sign a consent form to ensure they understood the information given and to also provide them opportunity to ask questions. The form also ensured participants were aware of their right to withdraw at any time. Participation in the study was completely voluntary with no incentives given. A plan was in place that if any road injury victims appeared distressed during interviews, or seemed in need of further support or information, they were to be referred to local disabled people’s organisations (DPOs) which were contacted prior to the interview process. This was however not necessary because none of the participants needed such support. It was not necessary to explain to any of the participants what mental health illness is and they all had a good understanding of the commonest symptoms.

### 2.5 Interview procedures and analysis

The interview script was informally piloted with five other research students prior to collecting data. Minor adjustments to wording and phrasing were made after this. All interviews were performed individually, at a time and place most convenient for the participants and conducted by the MC who was a doctoral student and a trained qualitative researcher. MC is also a clinician and had previous extensive experience of conducting interviews. The interviews were recorded and lasted 40 minutes on average. No repeat interviews were made due to time limitations and geographical constraints. Interviews continued until data saturation was achieved (14 for the interviews with RTI survivors and 20 for HCWs). Participation in the study was entirely voluntary without any monetary benefits or gifts were given.

Regarding the demographic characteristics of injury victims, 57% (n = 8), were male, 43% (n = 6) were female, 79% were aged below 40, with the average age of 34.6 years. Meanwhile of the HCWs interviewed, 55% (n = 11), were male, and 45% (n = 9) were female, with an average of 9.6 years’ work experience between them. An important characteristic of the sample was that t he HCWs involved in the study were all involved in caring for RTI patients through the MVAF and as such had valuable insights about how the MVAF system functions. They included rehabilitation, nursing, and medical professionals in different regions of the country, working in both the public and private sectors.

Semi-structured, open-ended questionnaires were developed for both interviews based on existing RTI questions and adapted for factors pertinent to RTIs, with a set of questions specifically focusing on access to psychological support as a component of these questionnaires. Questions included information on type of injury and costs associated with health care following the injury. The questionnaires also collected demographic and socio-economic information, including age and gender.

### 2.6 Analysis

Analysis commenced with careful review of tapes followed by full transcription of audio recordings. The lead author (MC) conducted all the transcriptions; these were done within seven days of the original interview. Transcripts were returned to participants to check for accuracy and resonance with their experiences and a few minor corrections were necessary.

Following the Braun and Clarke [23] six-step framework, data from both sets of interviews were analysed using thematic analysis. The framework is reportedly the most widely used approach in thematic analysis by providing a clear and usable framework for conducting analysis [24]. An important advantage of thematic analyses is that it not only allows for data interpretation, but is also beneficial for producing analyses suited for informing policy development [23]. The data was coded manually because of the relatively small sample size and no software was used. As noted earlier, this study was part of a PhD investigating the long-term impact of road injuries in Namibia. Thus the lead author did all of the coding. The coding is tree is available elsewhere [22]. In line with an inductive approach, we had no pre-defined themes to guide the coding process and the codes formulated were data-driven. A thematic map was developed to show the themes and how they related to each other [22].

## 3.0 Findings

The themes related to this specific study are outlined in the next sections:

### 3.1 Access to psychological support

Most of the injured survivors interviewed reported ongoing mental health issues related to the road injury regardless of the severity of injury and the length of time from the injury. Specifically, even those who had the least severe injuries reported these ongoing issues. Despite this, almost half of the participants had not received any psychological support or counselling following the road injury despite the fact that the majority of them stated that they felt they would have benefited from this. For example L7, a 37 year male employed in the military, sustained rib and foot fractures with soft tissue injuries and reported needing psychological support:

**Yeah, that’s what I’ve been saying that I needed, because these things, they are still running too much in my mind. I need some counselling.***[L7*, *37yr male]*

Contrary to this L14 had received psychological support but felt it had not been helpful. She felt the psychologists could not understand her situation and had found it better to talk to people who had also been injured in a road crash and sustained serious injuries. This had helped her to cope with the new situation. In her own words:

**The psychologists would come to me and I would just say; just go because you don’t understand. You keep telling me that you understand. So, as soon as I started this group whereby us in wheelchairs, ladies in wheelchairs. We talk and you know, it’s kind of a relief because you talk to people who are all in the same situation. So when that person says I understand, then you know that person understands. It’s the same with AA groups or whatsoever groups. When you talk to people facing the same situation, it’s much more of a relief. It’s much more that you can relate to that person, and through that, you can even counsel yourself because you can open up and speak more about it and realise you’re not the only person in the situation. So, it helped a lot to talk about it.***[L14*, *young female in Windhoek]*

On the other hand, L1, a 28-year-old female, had not received any psychological support but felt psychological support had not been necessary for her. She found the support from her family helped her greatly in coping with the injury. She also felt that the injury was part of God’s plan and that everything happens for a reason.

Contrary to the general trend, one of the wheelchair users (L3), a 33-year-old in Erongo, reported she felt well and the injury had no longstanding impacts on her mental health. She cited her family as having been very supportive. During her interview she reported she lived with her parents and her siblings were also involved in her life, with her brother driving her to most places and all employed family members giving her money on a regular basis.

Notably, the participants who described having the poorest mental health (L2 and L4) also reported having been abandoned by their families and not receiving any support from them. L4 reported receiving psychological and psychiatric care which helped initially in hospital, but once he was back home, his cycle of depression would manifest again due to poor family support.

Most HCWs felt psychological support was generally lacking in Namibia for RTI survivors despite the involvement of the MVAF paying for all health care of injury victims. HCWs reported that under the scheme, all injury survivors were eligible to have psychological treatment if this was deemed to be necessary by the HCW, who would then inform the patient’s MVAF case manager in order for this to be sanctioned. For example P11 reported that they rarely referred for such services and that the referral system was inadequate in terms of this:

**I’d want to say we are lacking in that area because we rarely ever send people for that type support. I had a lady that lost her kids in a car accident. Not even once did I remember referring to a psychologist. So I think we are lacking in that area. There is a need for improvement.***[P11*, *physiotherapist in Caprivi]*

Many HCWs highlighted that the major problem was a lack of psychologists and counsellors. P6, a senior public sector occupational therapist, revealed that there were only two psychologists working in the public health services in the whole country while P17, a private Occupational therapist practitioner, indicated that even in the private sector the psychologists were only available in the capital city, Windhoek, and not the rest of the country such that those outside the capital were strongly disadvantaged in accessing this compared to those in Windhoek. The participants highlighted that due to the lack of psychologists, social workers were the ones mostly involved in the counselling of road survivors. For example P2 worked in the spinal rehabilitation unit and discussed the counselling services there:

**In our unit we do have a social worker who deals with such cases. Here in Namibia, the social worker plays the role of being the counsellor.** *[P2]*

One of the participants had worked in several regional countries and expressed some surprise with the role of social workers as being counsellors to the road injury survivors. She however added that members of the whole team looking after the injured were also contributing in this.

**I don’t know, it’s only here that I found social workers being counsellors. The social workers are the ones who are chipping in as counsellors. But then, the whole multidisciplinary team also act as counsellors when you work with the patient.***[P6*, *Senior Occupational therapist]*

Several other participants also reported that they were contributing to trying to counsel the injured because of the lack of psychologists. A few participants also explained that psychological support was not necessary for everyone sustaining road injuries and reported that it was only when someone had been identified to need this that they would be referred.

### 3.2 Other coping mechanisms

The support of family friends was cited most by the participants as having helped them to cope with their injury. The second most common coping method mentioned was belief in God and going to church. One of the injury survivors, seriously depressed in the aftermath of the accident, reported that his faith in God had helped to prevent him from committing suicide:

**Without the Bible, I would have been dead already. I wanted to commit suicide, maybe like four or five times. You know, it’s not easy, everybody looks past you. It’s quite a heavy process.***[L2*, *27 year old male]*

Other ways of coping that were mentioned by participants included a wheelchair basketball team, and a wheelchair ladies support group, WhatsApp groups, and motivational speaking. Interestingly one of the participants (L11) highlighted one of the rehabilitation coaches employed by the MVAF as a source of inspiration and comfort. The rehabilitation coaches were individuals employed by the Fund to help the newly injured wheelchair users with spinal injuries adjust to their new situation. They had been injured in crashes themselves and were also wheelchair users.

**She is my hero in that sense. Because when I was in hospital, she gave us a testimony about how she got injured. She got injured at a young age but she kept on until she became independent. Yeah, she had a lot of challenges, but she overcame them. When you look at a young lady like that doing things like that, why not me? Yeah, she has empowered me more about the fact that I cannot give up. I must try my best to also become independent one day.***[L11*, *39year-old male]*

None of the participants reported other copying mechanism, such as drugs or alcohol following their road injury. This is however a sensitive area to broach and it is possible participants may not have felt comfortable to share such details.

## 4.0 Discussion

An objective of this study was to identify the levels of psychological support available to road injury survivors. As noted in Section 1.0, this has been reported to be an area lacking in research globally [16]. The few studies done, primarily in HICs have showed that psychological consequences from RTIs are often neglected by clinical staff treating patients despite the considerable emotional and psychological consequences [17]. Comparable studies from LMICs are almost entirely lacking.

The findings in this study correlate with these findings from HICs. Almost half the injury survivors had not received any counselling or psychological support, although most reported they felt they would have benefited from this. HCWs gave similar reports and noted the main reason for this was that although such services were free for road injury survivors requiring or referred for this, the services were often unavailable due to a severe shortage of psychologists and counsellors in Namibia. These reports by HCWs are congruent with the report given by [19] which highlighted that the delivery of mental health care services in the public health sector is particularly compromised by shortage of physical infrastructure, and trained and qualified professionals. This report highlighted that there are only two state mental health care departments, with the Ministry of Health and Social Services only employing five psychiatrists and three psychologists countrywide, a situation reported to severely hinder service delivery. This is also a common issue in LMICs. According to [25], empirically supported psychological treatments are among the most effective mental health interventions for treating mental health problems such as PTSD but are however not accessible for most populations in most countries. This is particularly true in LMICs, which have a shortage of professionals trained in mental health, resulting in a treatment gap of up to 93% [26]. In summation, given the acknowledged high burden of mental health problems following road injury, our results indicate that many of those injured in RTIs in Namibia are not receiving the psychological support they require and which could be covered by Namibia’s effective MVAF system. These individuals are at great risk of not reaching the optimal recovery possible which consequently impacts negatively on their overall quality of lives. The primary way of combating this problem is for the government of Namibia, and universities and colleges, to be lobbied to train more mental health professionals in order to extend services as well as ensuring that these workers are not concentrated in Windhoek, the capital city, such that services become more decentralised and can reach populations in other areas. To support this, shorter courses can also be introduced to train of auxiliary mental health staff with basic training in providing counselling services. This has been done successfully with physical rehabilitation in several LMICs such as India, Lebanon, Myanmar, Thailand, Viet Nam, and Zimbabwe, which have all responded to the lack of professional resources by establishing mid-level training programmes [27]. The advantage of mid-level training is that it is less expensive, and although it may be insufficient by itself, it may be an option for extending services in the absence of full professional training [28].

Additionally, with the aftermath of COVID-19 and the switch to on-line counselling, one potential option to quickly expand beyond Windhoek is for services to consider strongly how some counselling like this could be done at distance online as most people in Namibia now own mobile phones. According to the [29] the use of information and communication technologies is a resource that can enhance the capacity and accessibility of treatment measures by providing interventions remotely. These techniques have successfully enabled people in remote areas to receive expert treatment from specialists located elsewhere in vast areas including cardiac rehabilitation [30], speech and language therapy [31], and cognitive rehabilitation for people with traumatic brain injury [32], as well as training and support of health-care personnel computerized guidelines to help clinicians use appropriate interventions [29].

Another viable option could be task shifting. This is a process whereby specific tasks are moved, where appropriate, to health workers with shorter training and fewer qualifications in a specific area [33]. “By reorganizing the workforce in this way, task shifting can make more efficient use of existing human resources and ease bottlenecks in service delivery” [33: pg 7].

Our study shows a form of this is already in existence with the use of social workers as substitutes for psychologists in providing mental health care for injury survivors in Namibia. Although no evaluation has been done in Namibia to show this is effective, a review by [34] on the use of task shifting in LMICs found this to be a potentially viable option for increasing access to mental health care. Supporting this, a review by [35] exploring psychological treatments in LMICs by care professionals not trained in a field closely related to mental health such as social workers, midwives, auxiliary health staff, primary care doctors and nurses, found merit to this. They concluded that innovations in global mental health to improve access to psychological treatments by using low-cost and widely available staff with no psychology training was more effective than usual care [35]. Similarly [18] found that participants in their study repeatedly reported relying on physiotherapists for information and support following their injury and viewed them as having the taken the place of counsellors. Works by [15] adds that HCWs with no psychological training, such as occupational therapists, could also support adaptive self-regulation in injured people by assisting them to set realistic goals and recovery expectations in order to improve perceptions of well-being and to alleviate feelings of helplessness about the future. In order to enhance this current model in Namibia, other HCWs involved in care of road injury survivors could be trained to also provide counselling services. In addition, training programs could be introduced in Namibia to train community based mental health workers and thus enhance access to care for communities which have less access including rural ones. This model in Namibia is also one that could be used in other countries in SSA and other countries in low resource settings. Follow up evaluation and research of such initiatives could also add significantly to our global understanding of how to expand the provision of low-cost, effective services for RTI victims.

Having more trained counsellors available is not only part of the problem. The system would need to be revised to ensure regular and appropriate referrals. Many of the HCWs interviewed for this project reported that they rarely refer road injury victims to psychological services. The reason for this may be because they lacked both a general and specific awareness of the symptoms of mental illness. Better training in this area for HCWs would be appropriate here and is supported by previous research such as the work of [12], who report that frontline HCWs in outpatient clinics and health care services should be alerted to the recognition of psychological symptoms and importance of referral to appropriate services for road injury victims. Thus it will be beneficial for HCWs, not trained in psychology, involved in caring for road injury victims Namibia to receive such training. To enhance the recognition of these symptoms, [12] adds that considering that emotional state of many injury survivors, it may also be important if at the point of discharge RTI survivors are made aware that they may begin or continue to experience such symptoms and, if they do, be provided with information about where to seek help. Additionally, some of the RTI survivors reported feeling as though the psychological support had not helpful and the psychologist had not understood them. Considering the small numbers of mental health practitioners in Namibia, this could be related to the practitioners developing compassion fatigue. This can be defined as the burden in emotional and physical aspects of life felt by a counsellor out of their service to the people in need and is a global problem which has a serious effect on the professional and personal life of any counsellor [36]. In Namibia, considerations should be made about how mental health staff could be supported in the long-term.

An additional significant finding was that some of the participants who had received counselling/therapy reported they had not benefitted from psychological treatment but found peer support groups to be more helpful. We could not find any literature on this in SSA but there is data from HICs. In their systematic review, [37] reported that a wide range of evaluative studies found improved coping skills, reassurance and a sense of normalcy, reduction in isolation, information sharing, a better understanding of the experience and future, and in some cases, greater confidence in talking to physicians as a result of peer support amongst those burdened with illness. Work by [15] also reported that peer support can also can provide social support, promote resilience and coping strategies, and sharing of ideas while [38] reported that they can also help to cultivate adjustment and enhanced self-efficacy and adjustment following injury which can lead to improved outcome. In a review on peer support, [39] reported that, compared with professional intervention, peer support appears to more successfully promote hope and belief in the possibility of recovery; empowerment and increased self-esteem, self-efficacy and self-management of difficulties; and social inclusion, engagement and increased social networks. As such, there has been exponential growth in the employment of peer support workers in the US, Australia and New Zealand and UK [39]. Thus peer support can be utilised in Namibia to enhance the current lack in mental health services available to road injury victims.

## 5.0 Limitations

A limitation to this study is the relatively small sample size. Although such a sample size is not unusual for qualitative studies and we recruited participants, until data saturation, whose shared experiences were invaluable sources of information and evidence, their experiences cannot be generalised to the entire population of those injured in RTIs in Namibia. Additionally, most of the individuals interviewed had severe injuries. Even though those who are less seriously injured are still traumatised and can have resultant ongoing mental health complications, a follow up study of people less seriously injured will also be beneficial as they would also greatly benefit from such services and evidence showing this would could justify the financial need for training additional mental health workers.

## 6.0 Conclusion

As far as we are aware, this is the first study of its kind in Namibia or SSA. It adds to the global knowledge on this topic as well as bringing new knowledge from a low resource setting. Our study findings are in line with other global studies done in showing inadequate access to psychological support for injury victims. It is hoped the findings and recommendations suggested will be of use to Namibian policymakers, health services and the government in coming up with ways of enhancing access to much needed and lacking psychological support for the many people injured in RTIs in Namibia. Given Namibia’s successful MVAF, the potential of some available funding to provide such services may allow Namibia to generate a viable mental health support model that could be widely copied in many LMICs countries around the world to enhance psychological support for road injury victims. In order to enhance the dissemination, contacts at the MVAF will be directly approached with the findings.

## Supporting information

S1 Appendix MVAF registration process. (DOC)

S2 Appendix Benefits provided by the MVAF. (DOC)

S3 Appendix Semi-structured questionnaire for injured survivors. (DOC)

S4 Appendix Semi-structured questionnaire for HCWs and advocates. (DOC)

## Abbreviations

DPO: Disable People’s Organisation
HCWs: Health care workers
LMICs: Lower and Middle Income Countries
MVAF: Motor Vehicle Accident Fund of Namibia
MTPL: Motor vehicle third-party liability insurance
NAPPD: Namibian Association of People with Physical Disabilities
NFPDN: National Federation of People with Disabilities in Namibia
RTI: Road Traffic Injury
SSA: Sub-Saharan Africa
