# Supplementary material for: The availability of psychological support following road travel injuries in Namibia: A qualitative study
Source: PLoS One. 2021 Oct 1;16(10):e0258197. doi: 10.1371/journal.pone.0258197 (PMC8486108; doi:10.1371/journal.pone.0258197)
Supplement: S1 Appendix — (DOC) [file pone.0258197.s001.doc]

**List of Appendices**

Appendix 1: MVAF registration process

| - According to the legislation, in the event of a road crash, ideally, the MVAF call centre has to be notified, following which they will dispatch an ambulance. - Crash survivors are triaged at the scene of crash and individuals with moderate or serious injuries are admitted to either state or private healthcare facilities, with those with minor injuries treated and discharged. - The injured person is issued with an MVAF reference number, and an MVAF case manager undertakes hospital visits and issues a claim form for submission by the injured individual. - Once the injured person submits the claim form, the MVAF assesses the claim based on the individual’s injuries and processes payment of an injury grant if this has been deemed applicable. - When complete information has been provided, claims are supposed to be processed within 30 days.      - Again, according to MVAF rules, following an injury should follow up rehabilitation or additional care be needed a case manager is assigned the case, and he/she drafts and implements rehabilitation plans in consultation with the injured individual. - The case manager obtains progress reports from specialists/doctors managing the individual to assess whether they need further rehabilitation. - Based on this assessment, the claim is either closed or further rehabilitation is provided. |
| --- |

Source MVAF (2018)
