# Supplementary material for: The availability of psychological support following road travel injuries in Namibia: A qualitative study
Source: PLoS One. 2021 Oct 1;16(10):e0258197. doi: 10.1371/journal.pone.0258197 (PMC8486108; doi:10.1371/journal.pone.0258197)
Supplement: S2 Appendix — (DOC) [file pone.0258197.s002.doc]

**Appendix 2: Benefits provided by the MVAF**

| Benefit type |  |
| --- | --- |
| Medical Benefits | An individual injured in a road crash is eligible for up to N$1,500,000 which provides for medical treatment, injury management, and rehabilitation. |
| Injury Grant | The Fund provides an injury grant to the value of up to N$100 000. This is a cash grant that serves as compensation for injury for any injured person, with certain limitations and exclusions. |
| Funeral Grant | The Fund provides a funeral benefit to the value of N$7 000 for any person who dies in a road crash in Namibia. |
| Loss of Income | Loss of income may be claimed by a survivor of a road crash up to N$ 100 000, with certain limitations and exclusions. |
| Loss of Support | Loss of support may be claimed by a dependent of a deceased up to N$ 100 000, with certain limitations and exclusions. |

Source (MVAF, 2018)

For those who have been deemed after assessment to have serious injuries such as paraplegia or quadriplegia, home modifications are also made if they or their parents own the property they want to be adapted. They are also provided with a monthly caregiver allowance.
