# Supplementary material for: The availability of psychological support following road travel injuries in Namibia: A qualitative study
Source: PLoS One. 2021 Oct 1;16(10):e0258197. doi: 10.1371/journal.pone.0258197 (PMC8486108; doi:10.1371/journal.pone.0258197)
Supplement: S3 Appendix — (DOC) [file pone.0258197.s003.doc]

**Appendix 3: Semi-structured Questionnaire for injured survivors**

**Part A: Demographic Information**

| **Age** |  |
| --- | --- |
| **Gender** |  |
| **Level of education** |  |
| **Type of injury** |  |
| **Severity of injury** |  |
| **Current employment status** |  |
| **Pre-injury employment status and occupation** |  |
| **Area/Region** |  |

P**art B: Injury, hospitalisation, rehabilitation and costs**

1. When were you involved in an RTI?

2. Did you receive any emergency care?

2a. If so, can you tell me more about this?

3 Were you hospitalised?

3a. If so, how were you transported to hospital?

3b. For how long was the period of hospitalisation?

4. Following discharge, did you receive any follow-up treatment?

4a. If yes, can you tell me more about this?

5. Following the injury, did you receive any medical rehabilitation, either in hospital or once back in the community?

5a. If yes, can you tell me more about this and how many sessions of treatment you had ?

5b. If not, could you tell me why you did not have any medical rehabilitation?

6. Did you receive any post-trauma psychological care/counselling?

6a. If so, could you tell me more about this?

7. Did the injury require you to use any mobility aids?

8. If so, where you able to access these aids?

8a. If so, could you tell more about the process of acquiring them?

8b. If not, what were the reasons that that prevented you from being able to access them?

9. Following the injury, were any changes/adaptations required to be made to your home environment?

9a. If so, were these done?

9b. If yes, could you tell me more about the process?

9c. If not, why were the changes/adaptations not carried out?

10. Where there any costs related to the medical care and rehabilitation of your injury/ies?

11. If yes, could you tell me more about how these costs have been met?

**Part C: Health status and well-being**

1. Can you tell more about any changes to your health status since sustaining the injury?

2. How is your current health status in terms of pain levels, mental health, physical and overall well-being?

3. Has the injury had any effect on your ability to carry out ADLs such as personal care?

3a. If so, could you tell me more about this?

4. Has your ability to move around the community been affected by the injury such as walking, using public transport?

4a. If so, can you tell me more about this?

5. Has the injury had any effects on your lifestyle and social life?

5a. If so, could you tell me more about them?

6. Has the injury affected how you relate with family members, friends and the wider community?

6a. If so could you tell me more about this?

7. How have you coped with the injury?

8. Has the injury had any effect on your behaviour in terms of drinking or using recerational drugs?

**Part D: Employment, loss of income and vocational rehabilitation**

1. Were you employed/studying before the injury?

1a. If so, what type of work were you doing?

2.What is your current employment status?

3. Has the injury had any effects on your employment situation or prospects?

3a. If yes, could you tell me more about this and whether there have been any opportunities to train to do other types of work?

4. Has the injury had any effects on your personal and household income?

4a. If yes, could you please tell me more about this?

5. Did you need to sell any family property or take any loans following the injury?

5a. If yes, could you tell me more about this?

6. Following the injury, have you had any financial and/or social-welfare support?

6a. If so, could you tell more about it and the sources who have provided this?

6b. If not, could you tell me some of the reasons?

**Are there any other issues/comments regarding this subject that I haven’t asked about but you think is important for me to know?**
