# Supplementary material for: The availability of psychological support following road travel injuries in Namibia: A qualitative study
Source: PLoS One. 2021 Oct 1;16(10):e0258197. doi: 10.1371/journal.pone.0258197 (PMC8486108; doi:10.1371/journal.pone.0258197)
Supplement: S4 Appendix — (DOC) [file pone.0258197.s004.doc]

**Appendix 4: Semi-structured Questionnaire for HCWs and advocates**

**Part A: Demographic Information**

| **Age** |  |
| --- | --- |
| **Gender** |  |
| **Level of education** |  |
| **Profession** |  |
| **Number of years worked** |  |
| **Current position** |  |
| **Level of position** |  |
| **Area/Region** |  |

1. Could you tell me more about your professional background and job?

2. How long have you been with this organisation?

3. How is the organisation involved in road safety in Namibia?

4. Are you aware of any other organisations involved in road safety in Namibia?

4a. If yes, could you tell me more about these organisations and how they are involved in road safety?

**Part B: Magnitude of RTIs and access to rehab following injury**

1. Can you tell me about road-injuries in Namibia?

2. Are they regarded as a public health problem in Namibia?

2a. If so, could you tell me more about this?

3 Does your service provide any care to road-injury survivors?

3a. If so, could you tell me more about the severity and type of injuries that you mostly encounter?

4. Are you aware of any other services which provide rehabilitation to people with road-injuries?

4a. If so, could you tell me about these?

5. Could you tell me more about the proportions of road-injury survivors needing rehabilitation who do receive this in Namibia?

6. Are there any differences between rural and urban areas and regional differences?

6a. If so, could you tell me more about this?

7. Are there any gender and age differences?

7a. If so, could you tell me more about them?

8. Are you aware of any barriers that limit people from road injuries from accessing rehabilitation services?

8a. If so, could you tell me more about them?

**Part C: Aids, long-term impairment and disability**

1. Could you tell me more about the availability of gait aids and provision of household adaptations to those who require them flowing a road injury?

2. Once someone has been involved in a serious RTI, is there any long-term follow-up on their situation and condition?

2a. If so, could you tell me more about this?

3. Are there any statistics as to how many people sustain permanent impairments or become disabled due to RTIs each year?

3a. If so, could you tell me about these figures and how the information is collected?

3b. If not, could you be aware of the reasons this information is not available?

4. Are there any statistics as to the total proportion of people living with permanent impairment/disability due to RTIs?

4a. If so, could you tell me more about the levels?

5. Are you aware of any long-term impacts of RTIs on those who sustain permanent impairment/disability?

5a. If so, could you tell me more about them?

6. Are there any measures in place to support those who have sustained long-term impairments and permanent disability?

6a. If so could you tell me more about these?

6b. Could you also tell me about any regional differences or rural/urban differences if they exist?

**Part D: Employment, loss of income and vocational rehabilitation**

1. Do road-injuries have any impact on the employment situations/prospects of survivors?

1a. If yes, could you tell me more about this?

2. Do they have any effect on personal and household income?

2a. If yes, could you explain further?

3. Are there any vocational rehab services for those unable to maintain same jobs due to disability?

3a. If yes, could you tell me more?

**Part E: Psychological and social support**

1. Following road injury, do survivors receive any psychological support/counselling?

1a. If so, could you tell me about this?

2. Are there any social welfare schemes in place to support them and family members?

2a. If so, could you tell me about them?

**Are there any other issues/comments regarding this subject that I haven’t asked about but you think is important for me to know?**
